# Metabarcoding Analyses of Gut Microbiota of Nile Tilapia (*Oreochromis niloticus*) from Lake Awassa and Lake Chamo, Ethiopia

**DOI:** 10.3390/microorganisms8071040

**Published:** 2020-07-13

**Authors:** Negash Kabtimer Bereded, Manuel Curto, Konrad J. Domig, Getachew Beneberu Abebe, Solomon Workneh Fanta, Herwig Waidbacher, Harald Meimberg

**Affiliations:** 1 Institute of Food Science, University of Natural Resources and Life Sciences Vienna (BOKU), 1090 Vienna, Austria; konrad.domig@boku.ac.at; 2 Department of Biology, Bahir Dar University, Bahir Dar 6000, Ethiopia; gech13@gmail.com; 3 Institute for Integrative Nature Conservation Research, University of Natural Resources and Life Sciences Vienna (BOKU), 1090 Vienna, Austria; manuel.curto@boku.ac.at (M.C.); meimberg@boku.ac.at (H.M.); 4 MARE-Marine and Environmental Sciences Centre, Faculdade de Ciências, Universidade de Lisboa, Campo Grande, 1749-016 Lisboa, Portugal; 5 School of Food and Chemical Engineering, Bahir Dar University, Bahir Dar 6000, Ethiopia; solworkneh@gmail.com; 6 Institute for Hydrobiology and Aquatic Ecosystems Management, University of Natural Resources and Life Sciences Vienna (BOKU), 1090 Vienna, Austria; herwig.waidbacher@boku.ac.at

**Keywords:** diversity, gut microbiota, 16S metabarcoding, Nile tilapia

## Abstract

The Nile tilapia (*Oreochromis niloticus*) gut harbors a diverse microbial community; however, their variation across gut regions, lumen and mucosa is not fully elucidated. In this study, gut microbiota of all samples across gut regions and sample types (luminal content and mucosa) were analyzed and compared from two Ethiopian lakes. Microbiota were characterized using 16S rRNA Illumina MiSeq platform sequencing. A total of 2061 operational taxonomic units (OTUs) were obtained and the results indicated that Nile tilapia from Lake Chamo harbored a much more diversified gut microbiota than Lake Awassa. In addition, the gut microbiota diversity varied significantly across the gut region based on the Chao1, Shannon and Simpson index. The microbiome analyses of all samples in the midgut region showed significantly higher values for alpha diversity (Chao 1, Shannon and Simpson). Beta diversity analysis revealed a clear separation of samples according to sampling areas and gut regions. The most abundant genera were *Clostridium*_sensu_stricto and *Clostridium*_XI genera across all samples. Between the two sampling lakes, two phyla, Phylum Fusobacteria and Cyanobacteria, were found to be significantly different. On the other hand, six phyla (Actinobacteria, Bacteroidetes, Chloroflexi, Firmicutes, Proteobacteria and Cyanobacteria) were significantly different across gut regions. In this study, we found that all samples shared a large core microbiota, comprising a relatively large number of OTUs, which was dominated by *Proteobacteria*, *Firmicutes*, Cyanobacteria, Fusobacteria and *Actinobacteria.* This study has established the bases for future large-scale investigations of gut microbiota of fishes in Ethiopian lakes.

## 1. Introduction

The microbiota of the gut has a significant effect on the health status of the host. The microbial community enhances the health of the host by inhibiting the performance of pathogenic microbes [1] and improving the immune response [2]. Moreover, gut microbiota facilitates the degradation of non-digestible fibers and synthesizes essential compounds such as vitamins [3]. It was also shown to be involved in the development of gut morphology [4]. The microbial structure of the gut changes with the developmental stage of the host and constantly adapts to the current situation [5]. Understanding the related mechanisms is crucial for economical important species, especially in aquaculture activities, where productivity is dependent on the animal health.

Nile tilapia (*Oreochromis niloticus*) is one of most frequent fish species in the world used in aquaculture. Production is expected to increase further due to the good performance in culture and its resistance to various environmental conditions [6]. In tropical areas, production is possible throughout the year. In temperate climate, severe mortalities occur during winter, and hence cold tolerance is an economically important trait in Nile tilapia [7]. A current study suggests that the gut microbiome might contribute to the fish adaptation in extreme environments, in particular, lower temperature [8]. It had been shown that fish populations inhabiting different geographical areas with different elevations from sea level showed gut microbiota composition variations [9]. To determine differences between populations from cold and warm environments could therefore help to characterize temperature adaptation pattern in the species. As a first step, we describe the microbiome composition of Nile tilapia (*O. niloticus*) from two Ethiopian lakes that harbor native populations of the species at two different elevations.

In Nile tilapia, the gut ecosystem is composed by diverse microbial groups, most of them are strict anaerobes. Traditionally, this has been assessed by conventional culture-dependent techniques. With this method, *Aeromonas hydrophila*, *Aeromonas veronii*, *Burkholderia cepacia*, *Chromobacterium violaceum*, *Citrobacter freundii*, *Escherichia coli*, *Flavimonas oryzihabitans* and *Plesiomonas shigelloides* have been identified from the gut of mature Nile tilapia [10]. Moreover, *Virgibacillus pantothenticus*, *Bacillus cereus*, *Bacillus licheniformis*, *Enterococcus faecalis* and *Virgibacillus alginolyticus* were isolated from the gut of Mozambique tilapia (*Oreochromis mossambicus*) [11].

In the aquatic environment, factors such as dissolved oxygen, temperature and salinity could affect the microbial structure in the gut of fish [12]. Moreover, trophic level and taxonomy of fish species can have an effect on composition of gut microbiota [13]. Nguyen et al. [14] reported that environmental parameters associated with seasons and geographic locations are among the factors that affect gut microbiota among fishes. The application of molecular approaches based on sequence diversity of the 16S ribosomal RNA gene helped to explore the gut microbiota more thoroughly, compared to the traditional cultural-techniques [15]. As determined by this sequence-based method, the dominant microbial group in most fish species is γ-proteobacteria [16,17]. The gut of East African cichlid fishes harbored *Fusobacterium*, Firmicutes and Proteobacteria [18]. A study on *O. niloticus* from Lake Nasser in Egypt revealed that cyanobacteria, alpha proteobacteria and methanogenic uncultured euryarchaeota were the dominant microbial groups [19].

Ethiopia is a country with a vast area of inland water bodies consisting of diverse aquatic ecosystems. There are more than 30 lakes in Ethiopia and the majorities are located in the rift valley region. The Ethiopian rift valley is a densely populated area with various agroindustry enterprises and mechanized irrigation farms. At the same time, it is one of the most environmentally vulnerable areas in the country [20]. Diverse groups of economically and ecologically important species of fish are dependant on the lakes. Lake Awassa is the prominent freshwater lake located in the central Ethiopian Rift Valley at the highest topographic position. It is near the city of Awassa, and has long been exposed to anthropogenic impacts, including over-fishing, irrigation, deforestation, overgrazing and indiscriminate use of pesticides and fertilizers in the catchment areas [21]. Several varieties of fishes are found in the lake of which the native African species, Nile tilapia (*O. niloticus*) is the dominant [22]. Lake Chamo is one of the lakes from the East African rift system in Ethiopia. The lake is rich in various species of fishes including Nile tilapia (*O. niloticus*), Nile perch (*Lates niloticus*) and tiger fish (*Hydrocynus forskahlii*) [23].

The studies in Ethiopian lakes focus mainly on diversity, feeding and migration related to the spawning habit of the fish [24,25,26]. The microbial diversity of fish gut in Ethiopia remains largely unexplored, and it is not known whether these fish species harbor unique gut microbiota or not. The aim of this study was to gather baseline data on the microbial complexities across the gut regions, and sample types (luminal contents and mucosa associated) of Nile tilapia (*O. niloticus*), one of the widely used aquaculture species of fish around the world. In addition, the gut microbial dynamics of samples of Lake Awassa and Lake Chamo were compared to collect first insights on environmental influence on microbiome composition.

## 2. Materials and Methods

### 2.1. Description of the Specimen Collection Sites

**Figure 1:**
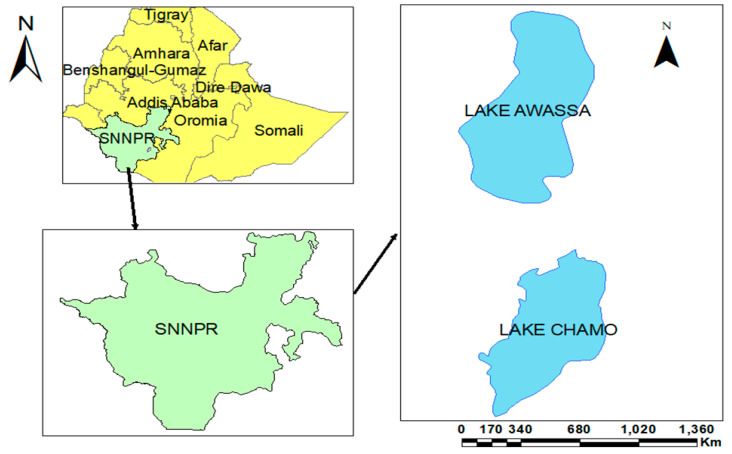
Study area. SNNPR: South Nation Nationality Peoples Region.

The specimens were collected in July 2018 from Lake Awassa and Lake Chamo. Lake Awassa is located between 06°58′ to 07°14′ N latitudes and 38°22′ to 38°28′ E longitudes, with an elevation of 1685 m above sea level (m.a.s.l) and it is the highest in altitude among the Rift Valley closed basin lakes. The maximum depth of the lake is 22 m. Its average pH is 8.17. The lake has an average temperature of 24.5 °C and its dissolved oxygen, total dissolved solid, nitrate and sulfate values of the lake water are 5.48, 974.5, 3.86 and 119.7 mg/L, respectively [21]. Lake Chamo is found in southern Ethiopia, and it is located in the Great Rift Valley at an elevation of 1235 m (a.s.l). The specific site lies between the coordinates of 5°50′0′′ to 5.83′33″ N latitude and 37°33′0″ to 37.55′ E longitudes. It is the southernmost lake of the Ethiopian Rift Valley (Figure 1). The temperature of the surface water is 28.58 °C and has an average pH of 8.66. The dissolved oxygen, total dissolved solid, concentration of nitrite and sulfate are 7.82, 725.58, 46.10 and 69.45 mg/L, respectively [27].

### 2.2. Fish Sampling and Processing

A total of 95 adult male Nile tilapia samples (45 from Lake Awassa and 50 from Lake Chamo) were purchased from the fishermen at the landing sites of Lake Chamo and Lake Awassa and sacrificed using high doses of clove oil [28]. Male individuals were used because of their larger size, facilitating the analysis and not to deplete female stock. Before dissection, fish samples and instruments were rinsed with 70% ethanol and aseptically dissected. The entire gut was removed and divided into three regions (stomach, midgut and hind gut). The gut luminal contents were collected following the procedure of Ghanbari et al. [29]. Gut contents were gently squeezed and placed in sterile screw cap tubes containing sterile phosphate-buffered saline and glycerol in equal volume. After collecting gut contents from the three regions, each part was thoroughly washed by soaking into 0.85% (*w/v*) saline solution. The gut mucosa-associated bacteria were collected as described before [30,31]. Gut mucosal samples were similarly placed into screw cap tubes containing phosphate-buffered saline and glycerol. Both luminal contents samples and mucosal samples were stored at −20 °C until further processing.

### 2.3. DNA Extraction

DNA extraction of gut luminal and mucosal samples were performed using the PowerFecal^®^ DNA Isolation Kit (Qiagen, Hilden, Germany) with some modifications. The modifications include heating the tubes at 70 °C instead of 65 °C after addition of C1 solution and using warm elution buffer preheated at 40 °C at the end. The final elution volume was 50 µL. The extracted DNA was stored at −20 °C until further processing. The quantity of the extracted DNA was checked using the Qubit dsDNA HS Assay Kit (Invitrogen, Carlsbad, CA, USA).

### 2.4. PCR Amplification and Sequencing

The amplicon sequencing approach was performed on a total of 95 individual DNA samples using the Illumina MiSeq system (Illumina, San Diego, CA, USA). DNA sequencing libraries targeting the V3–V4 hypervariable region of the 16S rRNA gene were prepared using the dual index approach from Shokralla et al. [32]. This consists of two PCR steps. In the first one, specific primers were used, while in the second, index information for sample identification was added. The first PCR was conducted with the primers 347F and 803R from Nossa et al. [33] extended with part of the Illumina P5 (TCTTTCCCTACACGACGCTCTTCCGATCT) and P7 (CTGGAGTTCAGACGTGTGCTCTTCCGATCT) adapters. PCR was performed in 10 µL reactions containing 5 μL of QIAGEN Multiplex PCR Master Mix (Qiagen, Hilden, Germany), 1 μL of each primer (1 μM) and 4 μL of template/genomic DNA. PCR was conducted using the following temperature profile: 95 °C for 15 min; 30 cycles of 95 °C for 30 s, 55 °C for 1 min, and 72 °C for 1 min; and a final extension at 72 °C for 10 min. PCR products were purified by mixing four microliters of PCR product with 2.9 μL of AMPure XP beads (Beckman Coulter Inc., Bree, CA, USA) and letting them incubated for 5 min at room temperature. Bound DNA beads were captured by an inverted magnetic bead extraction device, VP 407-AM-N (V&P Scientific, INC., San Diego, CA, USA) and washed twice in an 80% 200 μL ethanol solution for 45 s. Later, the beads were dried at room temperature for 5 min and eluted in 17 μL of elution buffer (65 °C 10 mM Tris-Hcl, pH 8.3). For index PCR, we used the TrueSeq apater sequences: P5: AATGATACGGCGACCACCGAGATCTACAC [Index] ACACTCTTTCCCTACACGACG; and P7:CAAGCAGAAGACGGCATACGAGAT [Index] GTGACTGGAGTTCAGACGTGT).

The PCR was conducted in a total volume of 10 μL containing 2 μL of each primer (1 μM), 5 μL of QIAGEN Multiplex PCR Master Mix and 1 μL of purified PCR product. The reaction was carried out, after an initial denaturation and activation at 95 °C for 15 min, using 10 cycles of 95 °C for 30 s, 58 °C for 60 s, and 72 °C for 60 s. All resulting products were pooled and sequenced using an Illumina MiSeq paired-end (PE) 300 sequencing platform (San Diego, CA, USA). The run was done as a joint run together with other libraries. About 10% of the reads should account to the microbiome pool. Sequencing was performed at the Genomics Service Unit, Ludwig-Maximilian’s-Universität München, Germany.

### 2.5. Sequencing Data Analysis

Sequences were quality controlled with Cutadapt v. 0.11.1 [34] by removing regions matching the adapter sequences and the remaining downstream sequence with the default settings. Regions with low sequence quality were excluded with the same program with sliding window approach allowing a minimum quality of 30. Trimmed reads with length below 200 bp were excluded. Paired reads were merged with PEAR v. 0.9.4 [35] with the default settings and deleting overlapped sequences smaller than 200 bp. Merged reads were checked if they contained the correct primer sequence information with an in-house script presented in Curto et al. [36] with small modifications. A maximum of two mismatches between primer sequence and read was allowed and matching regions were trimmed out. USEARCH 6.0 was used to further detect chimeras based on the RDP pipeline [37]. Usearch global alignment algorithm was applied to achieve operational taxonomic units OTU table by mapping high-quality reads to the remaining OTUs at 97% cutoff. Data filtering was done using default minimum count of 4 and 20% prevalence on MicrobiomeAnalyst in order to remove low quality or uninformative features [38]. Data rarefaction to minimum library size was done before further downstream processing.

Alpha diversity of each sample was assessed using the Chao1, Shannon and Simpson index. Beta diversity was determined based on the Bray-Curtis index distance method and principal coordinate analysis (PCoA) plots were made. In addition, non-metric multidimensional scaling (nMDS) was generated. Permutational multivariate analysis of variance (PERMANOVA) was used to analyze beta diversity. A one-way ordered analysis of similarity (ANOSIM) and homogeneity of group dispersions (PERMDISP) tests were also conducted on Bray-Curtis index distance method to supplement results of the PERMANOVA using MicrobiomeAnalyst [38]. The statistical significance of gut microbiota structure between different sampling sites and gut regions was assessed by non-parametric univariate Mann-Whitney/Kruskal-Wallis test. According to Turnbaugh et al. [39], the core microbiome is defined as the minimum community of microbes that is essential for the good functioning of the ecosystem. The core microbiome analysis was done as described in MicrobiomeAnalyst [38]. To detect the core microbiome, 20% prevalence and 0.01% relative abundance was used. Linear discriminant analysis effect size (LEfSe) were used to identify significantly different abundances of bacterial taxa across all samples. The analysis first performs non-parametric factorial Kruskal-Wallis (KW) sum-rank test to detect features with significant differential abundance with respect to the class of interest, followed by linear discriminant analysis to estimate the effect size of each differentially abundant features.

## 3. Results

**Figure 2:**
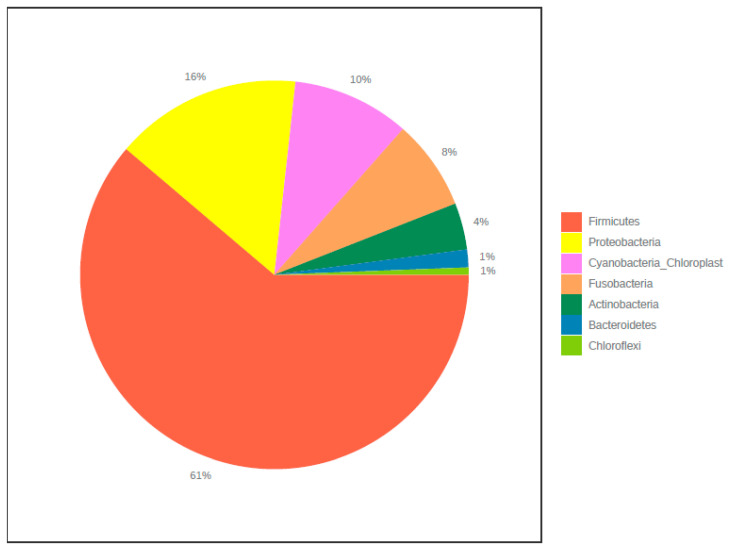
Microbiome composition of all samples at phylum level.

In total, 849,199 raw reads were obtained for the library pool of Nile tilapia microbiome after sequencing. After the initial quality filtering process, 718,091 sequences were retained. This resulted in a mean read depth per sample of 1930 sequences. In all samples, a total of 2061 OTUs were detected. Seven phyla and 41 genera were detected. The majority of sequences belonged to members of Firmicutes (61%) and Proteobacteria (16%) (Figure 2). Other phyla including Bacteroidetes and Chloroflexi were less represented. Rarefaction curves showed that plateau level was reached in all samples (Supplementary Figure S3). All samples have a Good’s coverage of more than 98% (Table S3).

### 3.1. Diversity Measures

**Figure 3:**
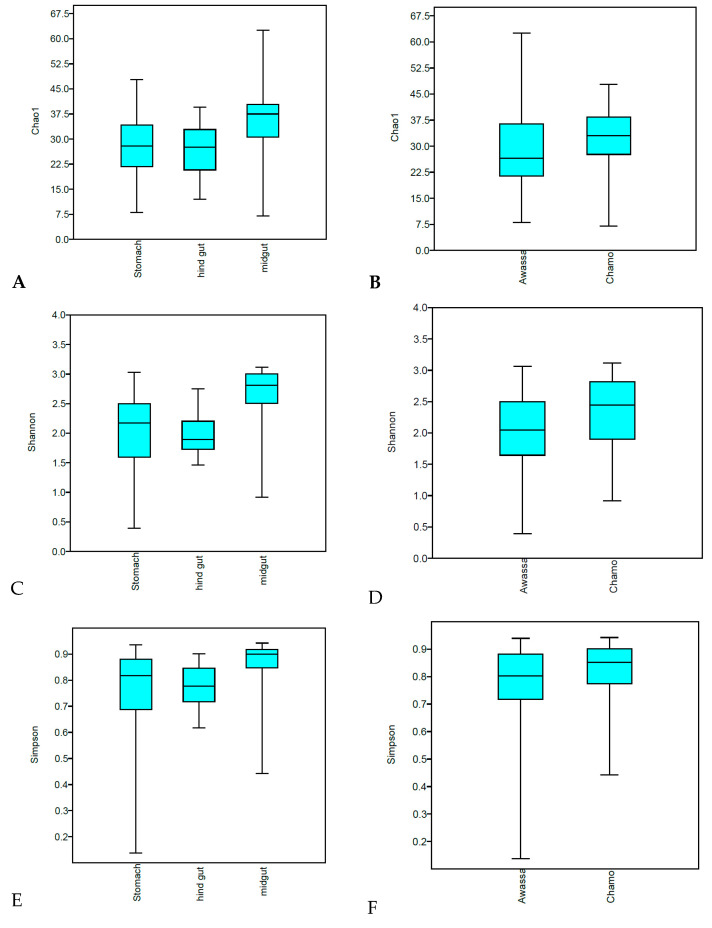
Alpha diversity of gut microbiome from Nile tilapia. (**A**) Chao1 of the gut regions. (**B**) Chao1 of the two lakes. (**C**) Shannon index of the gut regions. (**D**) Shannon index of the two lakes. (**E**) Simpson index of the gut regions. (**F**) Simpson index of the two lakes.

**Figure 4:**
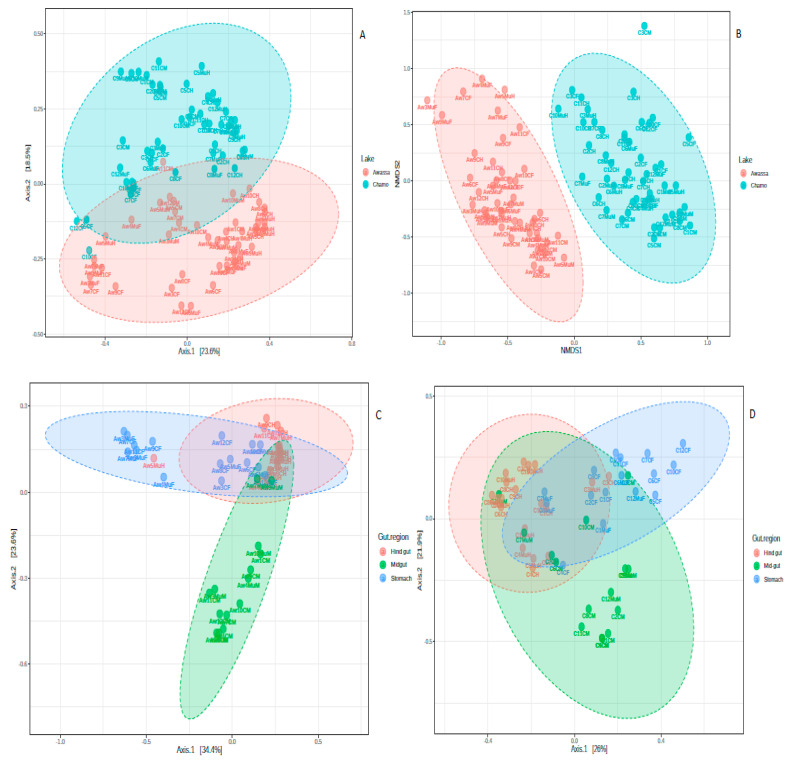
Principal coordinate analysis (PCoA) and non-metric multidimensional scaling (NMDS) based on the Bray-Curtis index distance method. (**A**) PCoA plot of all samples based on the two lakes. (**B**) NMDS plot of all samples based on the two lakes. (**C**) PCoA plot of samples from Lake Awassa. (**D**) PCoA plot of samples from Lake Chamo. Each dot represents one sample.

The result of alpha-diversity clearly indicates Nile tilapia from Lake Chamo harbored a much more diversified gut microbiota than Lake Awassa (*p-value 0.009*, *0.008* and *0.025* for Chao1, Shannon and Simpson indexes, respectively) (Figure 3). In addition, gut microbiota diversity varied significantly across the three gut regions, with *p-values 0.006*, *0.008* and *0.03* for Chao1, Shannon and Simpson indexes, respectively. The midgut had a higher diversity than stomach and hind gut. Beta diversity analysis revealed a clear separation of samples according to sampling areas and gut regions (Figure 4). Non-metric multidimensional scaling (nMDS) showed a close association between the samples from one lake, while both lakes were differentiated from each other. A close association was shown between samples of one gut region (Figure 4). Furthermore, statistical analysis of beta diversity across samples showed significant divergence of the microbial communities across fish sampling sites and gut regions (ANOSIM tests *p < 0.001*, *R: 0.4*). In addition, the non-significant results of the PERMDISP test of the two sampling lakes (*p-value = 0.967*) indicated that the results of the PERMANOVA can be interpreted as true differences in the location of samples or the average community composition. Besides, a significant PERMIDISP result (*p-value 0.0003*) and PERMANOVA (*p-value < 0.001*) of the sampling gut regions indicates there is a strong dispersion effect (variability in the community composition). On the other hand, no significant differences were detected between the microbial communities of intestinal luminal content and mucosa-associated microbiota (Supplementary Figure S2).

### 3.2. Core Microbiome

**Figure 5:**
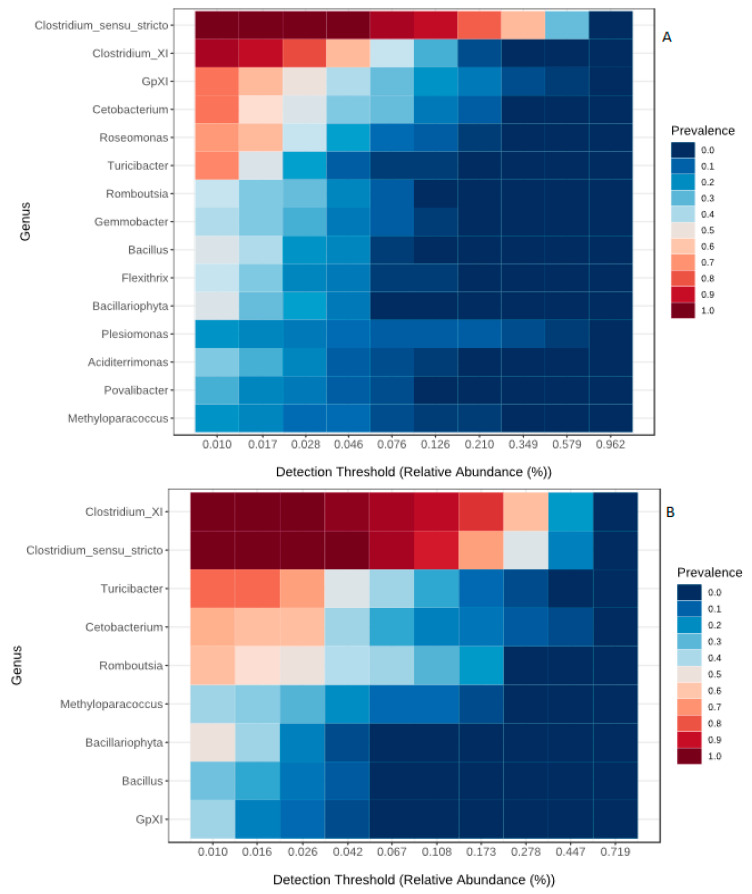

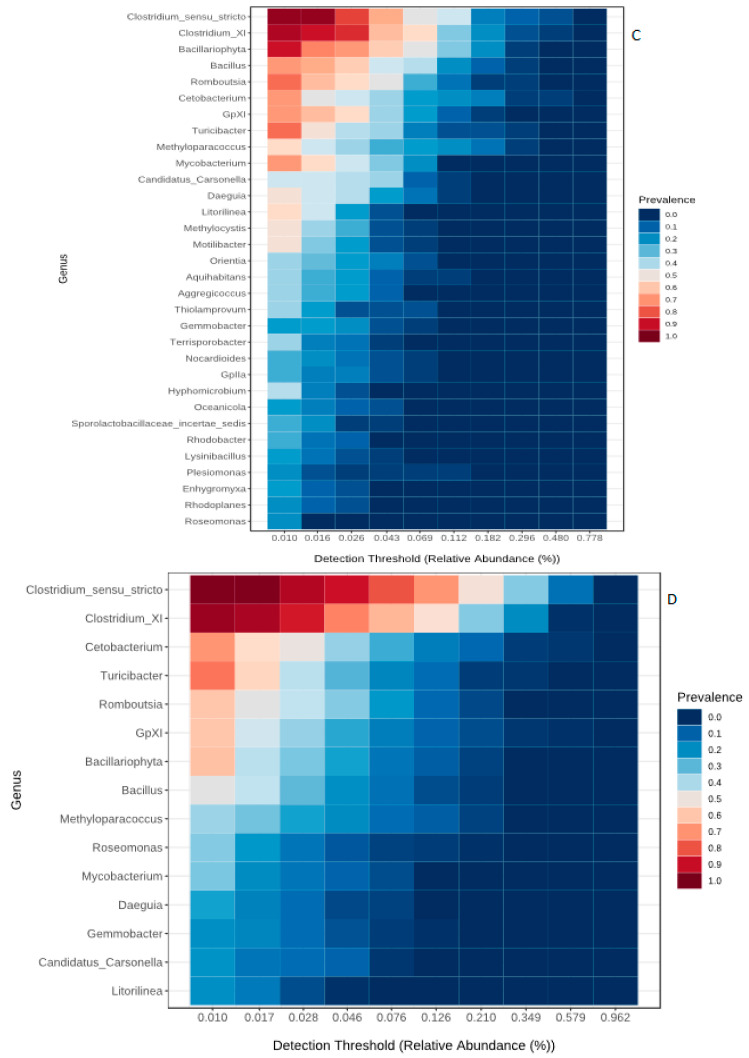
Heatmap representing the core microbiome at Genus level. Core microbiome at stomach region (**A**), hind gut region (**B**), midgut region (**C**) and the shared microbiome among the regions (**D**).

The core microbiome of the present study comprised of six phyla, 14 families and 15 genera (Table 1). Three families, Nannocystaceae, Nocardioidaceae and Sporolactobacillaceae, and five genera, *Sporolactobacillaceae_incertae_sedis*, *Rhodobacter*, *Nocardioides*, *Oceanicola* and *Enhygromyxa* were restricted to Lake Chamo only. Of the six core phyla, Firmicutes was the most abundant from all samples. At the family level, Clostridiaceae_1 and Peptostreptococcaceae were found to be the most dominant. *Clostridium*_sensu_stricto and *Clostridium_XI* were the most dominant taxa across all samples. The core gut bacteria of stomach dominated by *Clostridium*_sensu_stricto, *Clostridium_XI*, GPXI, *Cetobacterium* and *Turicibacter* (Figure 5A). Additionally, the bacterial genera *Clostridium*_sensu_stricto, *Clostridium*_XI, GPXI, *Cetobacterium*, *Turicibacter*, *Bacillariophyta*, *Bacillus*, *Romboutsia*, *Mycobacterium* were also found to be the dominant core bacteria in the midgut region (Figure 5C) and *Clostridium*_sensu_stricto, *Clostridium*_XI, *Cetobacterium* and *Turicibacter* in the hind gut region (Figure 5B). The genera *Flexithrix*, *Aciditerrimonas* and *Povalibacter* were exclusive for stomach. Twenty genera including *Mycobacterium*, *Daeguia*, *Litorillinea*, *Methylocystis* and *Motilibacter* were exclusive for mid gut region. The overall core gut bacteria of all gut regions consisted of 15 genera (Figure 5D).

### 3.3. Differential Abundance Analysis

**Figure 6:**
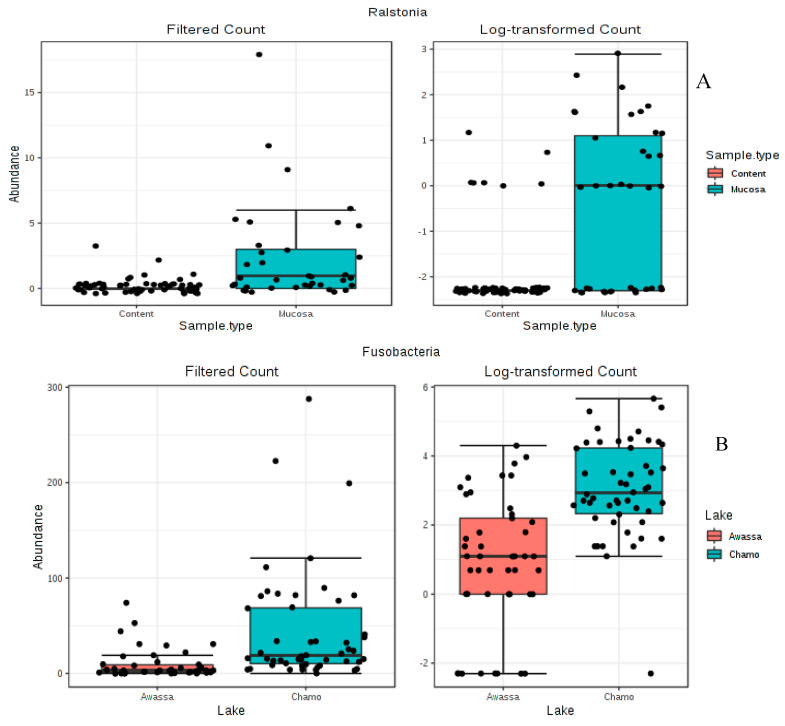

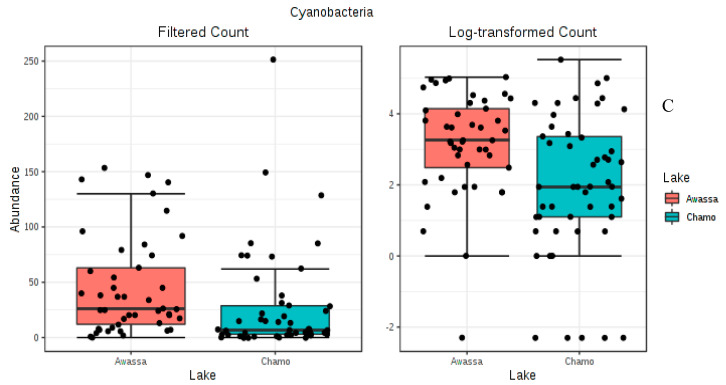
Differential abundance analysis done by a nonparametric test (Mann–Whitney/the Kruskal–Wallis test) as it is implemented in MicrobiomeAnalyst [38]. (**A**) Differential abundance analysis of the Genus *Ralstonia*. (**B**) Differential abundance analysis of the Phylum Fusobacteria. (**C**) Differential abundance analysis of the Phylum Cyanobacteria.

Differential abundance testing between all samples were done by a nonparametric test (Mann–Whitney/the Kruskal–Wallis test) as it is used in MicrobiomeAnalyst [38]. The result of this analysis clearly indicated significant difference for only two phyla, Phylum Fusobacteria (*p-value = 1.226E−7*) and Cyanobacteria (*p-value = 0.0013*) (Figure 6) between the two lakes. On the other hand, six phyla (Actinobacteria, Bacteroidetes, Chloroflexi, Firmicutes, Proteobacteria and Cyanobacteria) showed significant variation across gut regions (*p-value < 0.05*) (Supplementary Figure S1). At genus level, a total of 33 and 34 significant genera were found between the lakes (Awassa and Chamo) and gut regions (stomach, mid gut and hind gut) respectively (*p-value < 0.05*) (Supplementary Tables S1 and S2). However, the genus *Ralstonia* was the only one to show significant variation between sample types (luminal content and mucosa) (*p-value < 0.05*) (Figure 6). Moreover, the abundance of *Ralstonia* was higher in mucosal samples than luminal content.

**Figure 7:**
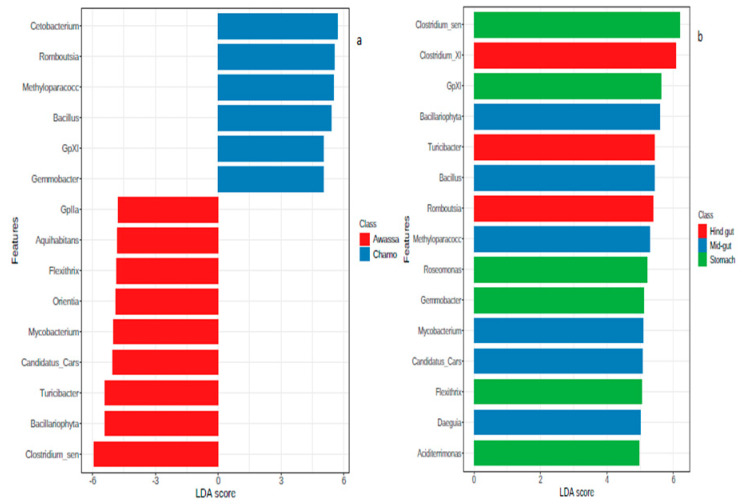
Linear discriminant analysis (LDA) effect size (LEfSe) for the two lakes (**a**) and the gut regions (**b**). Horizontal bars represent the effect size for each taxon.

Linear discriminant analysis (LDA) effect size (LEfSe) of the gut region showed *Clostridium*_sensu_stricto was the taxa contributing most to the dissimilarity of the stomach. On top of this, *Bacillariophyta*, *Bacillus* and *Methyloparacoccus* contributed most for the mid gut. Moreover, *Clostridium* XI was found to be the most contributing for hind gut. LEfSe analysis revealed significant bacterial differences between samples from Lake Awassa (negative scores) and Lake Chamo (positive scores) (Figure 7).

## 4. Discussion

The bacterial composition along the gut of Nile tilapia of Lake Awassa and Chamo was analyzed using high-throughput sequencing of the 16S rRNA genes. Fish from Lake Awassa showed lower microbial diversity than fish from Lake Chamo, which might be due to high pollution load from the municipality, regional hospital and factories surrounding the lake [40]. A recent study conducted on the water quality status of Lake Awassa reported that the concentration of metals such as manganese (0.83 mg/L), zinc (5.75 mg/L), chromium (0.22 mg/L), phosphate (1.31 mg/L) and biochemical oxygen demand 5 (BOD5, 68.7 mg/L) exceeded the WHO standard [21]. The effect of pollutants and toxins present in the environment on gut microbiota of fish was reported by several authors [41,42]. The composition of intestinal microbiota was changed following copper exposure in common carp [42]. High throughput sequencing of the 16S rRNA gene V3–V4 region revealed a significant change in the richness and diversity of microbiota in the gut of polystyrene MP-exposed zebrafish [41]. Moreover, the microbial diversity differences observed in this study might be due to the availability of feed sources in the lakes since feeding habits can greatly influence the structure and composition of the gut microbiota [43].

In this study, the gut microbiota diversity varied significantly across the gut regions (stomach, mid gut and hind gut) as determined by the Chao1, Shannon and Simpson index. Besides, beta diversity analysis revealed a clear separation of samples in accordance to these gut regions (Figure 4) and a closer association between samples of the same gut region. Similar results are reported in other studies. The gut microbiota in porcine, for example, exhibited significant differences in various intestinal segments [44]. The physiological differences between various segments of the gut of piglets indicated significant microbiome composition divergence [45]. The present study indicates that the midgut had a higher diversity than stomach and hind gut. Though no definitive distinction exists between the midgut and hind gut in Nile tilapia, the mid gut is the longest portion of the gut, which extends from the stomach to the posterior part of the gut. This region is the location where majority of digestive activities occur and believed to have consortia of microbes involved in digestion [46].

This study depicted no significant difference in the alpha and in beta diversity between intestinal content microbiota and mucosa-associated microbiota (Supplementary Figure 2). This contradicts previous studies where significant variations between luminal and mucosal microbiota was reported [47,48]. This study is the first to explore the variations of mucosal and luminal microbiota of Nile tilapia gut.

The dominant phylum in the gut of Nile tilapia in the present study was Firmicutes followed by Proteobacteria. This result agrees partially with previous studies done elsewhere on Nile tilapia. In the gut of genetically improved farmed tilapia, Proteobacteria, Firmicutes and Cyanobacteria were the most dominant phyla reported [49]. Moreover, Ran et al. [50] reported that Fusobacteria, Proteobacteria and Bacteroidetes were the dominant groups of microbiota from the gut of Nile tilapia. Fusobacteria, Bacteroidetes and Proteobacteria were also reported from the gut of Nile tilapia as a dominant phylum in study by Ray et al. [51].

To elucidate the ecology of gut microbiome fully, identifying the core microbiome is needed and it is the first step in defining a ‘healthy’ community [52]. In this study, we found that all samples shared a large core microbiota. The core microbiota was dominated by Proteobacteria, Firmicutes, Cyanobacteria, Fusobacteria, Actinobacteria and Chloroflexi. A core gut microbiota has been reported for certain fish species; such as rainbow trout [53], Atlantic cod [54], Atlantic salmon parr [55], zebrafish [56] and cichlid fishes [18]. In cichlid fishes studied; Firmicutes, Fusobacteria, Proteobacteria, Bacteroidetes, Actinobacteria, Planctomycetes and Verrucomicrobia were reported as predominant phyla. Therefore, this study agrees with the study on cichlid fishes. Identification of unique taxa such as *Flexithrix*, *Aciditerrimonas* and *Povalibacter* in the stomach and 20 genera in the midgut region indicates host physiological selection. The detection of a core microbiota for the three gut regions suggests that these bacteria are capable of colonizing the different anatomical regions.

The most abundant core OTUs at the genus level were *Clostridium_sensu_stricto*, *Clostridium_XI*, *Turicibacter* and *Cetobacterium*. All these genera have been previously reported as part of gut microbiota of fishes [57]. *Clostridium* is associated with cellulose degradation in the gut of freshwater fishes and *Cetobacterium* is involved in the degradation of protein in carnivorous fish [57]. Since Nile tilapia is omnivorous fish and capable of eating both, cellulosic materials and animals in particular zooplanktons, it is expected to find the predominance of *Clostridium* and *Cetobacterium* in their gut. *Cetobacterium* also involved in the production of Vitamin B12 [58,59]. Since Vitamin B12 acts as a modulator of gut microbial ecology [60], the abundance of *Cetobacterium* might be essential for having healthier gut microbiota. In addition, *Cetobacterium* spp. promotes decomposition of consumed organic debris, phytoplankton or zooplankton [61].

Many taxa were found to be differentially abundant between the different sections of gut and the two lakes as determined by univariate Mann-Whitney/Kruskal-Wallis test and LEfSe test. Between the two sampling lakes, only phylum Fusobacteria and Cyanobacteria showed significant differences. Besides, LEfSe analysis also revealed significant bacterial differences between samples of Lake Awassa and Lake Chamo (Figure 4). Similarly, Candis et al. [51] identified Fusobacterium at significantly higher levels in the gut of Nile tilapia. Moreover, Fusobacteria-like OTUs were reported from fish gut as a core microflora [62]. On the other hand, Cyanobacteria was reported as a major component of gut microbiome from the gut of Nile tilapia in Egypt [62].

In our study, Actinobacteria, Bacteroidetes, Chloroflexi, Firmicutes, Proteobacteria and Cyanobacteria were significantly varied across gut regions (stomach, mid gut and hind gut) (*p*-value < 0.05). Most of the gut microbial studies on Nile tilapia were focused on the posterior gut and to the best of our knowledge, this is the first report which covers stomach, mid gut and hind gut. However, similar results were reported elsewhere from the gut of other fishes and a particular region of Nile tilapia. Actinobacteria, Protebacteria and Firmicutes reported to be the dominant phyla throughout the whole gastrointestinal tract of Gilthead Sea Bream (*Sparus aurata*) [63]. The cyanobacteria *Microcystis* spp found to be the dominant microbiota in the stomach of Nile tilapia [64]. LEfSe analysis of the gut regions showed the taxa contributing most to the dissimilarity of the stomach to be *Clostridium*_sensu_stricto. Moreover, for the mid gut, *Bacillus*, *Bacillariophyta* and *Methyloparacoccus* and for hind gut *Clostridium* XI were found the most contributing. Bacterial diversity in the posterior gut sections of temperate marine herbivorous fish species from New Zealand were dominated by members of clostridial clusters XI and XIVa [65]. Protease and cellulase producing bacterial strains were found in large number in hind gut whereas highest number of amylolytic bacteria were found in foregut regions of *O. mossambicus* (Peters) and *O. niloticus* (Linnaeus) [66]. Similarly, amylolytic bacteria were also isolated from the foregut of Jundiá Catfish [67]. Since Clostridium is a cellulose degrader, our result agrees to their findings. In our study, the genus *Ralstonia* was significantly varied between sample types (content and mucosa) (*p*-value < 0.05) (Figure 5) and its abundance is higher in mucosal samples than content. Although studies on the gut mucosal microbiota in Nile tilapia are lacking (to the best of our knowledge), *Ralstonia* as a dominant mucosal intestinal microbiota was reported from sea bass [68]. In another study, the dominant autochthonous bacteria in the GI tract of yellow grouper belonged to Proteobacteria [69].

## 5. Conclusions

This study characterizes bacteria associated with the gut of Nile tilapia from Lake Awassa and Lake Chamo using the 16S rDNA metabarcoding technique. The diversity of bacteria associated with the gut of Nile tilapia collected in our study varied both between the two lakes and sampling gut regions. The observed differences in microbial compositions may be due to different selection pressure occurring in these environments and are likely to have different physiological implications. In this study, we found that all samples shared a large core microbiota, comprising a relatively large number of OTUs, which was dominated by Proteobacteria, Firmicutes, Cyanobacteria, Fusobacteria and Actinobacteria. The study has established the bases for future large-scale investigations of the gut microbiota of fishes in Ethiopian lakes. In order to make assumptions about the ecological consequences of microbiome composition, a much deep and large-scale investigation of the gut microbiota of Nile tilapia from more lakes in the region is also necessary.

## Figures and Tables

**Table 1:** List of the core microbiota.

Phylum	Class	Order	Family	Genus
Firmicutes	Clostridia	Clostridiales	Clostridiaceae_1	*Clostridium*_sensu_stricto
			Peptostreptococcaceae	*Clostridium_XI*
				*Romboutsia*
	Bacilli	Bacillales	Bacillaceae 1	*Bacillus*
	Erysipelotrichia	Erysipelotrichales	Erysipelotrichaceae	*Turicibacter*
Cyanobacteria	Cyanobacteria	Family XI	Family XI	*GpXI*
	Chloroplast	Chloroplast	Chloroplast	*Bacillariophyta*
Fusobacteria	Fusobacteriia	Fusobacteriales	Fusobacteriaceae	*Cetobacterium*
Proteobacteria	Gammaproteobacteria	Methylococcales	Methylococcaceae	*Methyloparacoccus*
		Gammaproteobacteria_incertae_sedis	Candidatus Carsonella	*Candidatus Carsonella*
	Alphaproteobacteria	Rhodospirillales	Acetobacteraceae	*Roseomonas*
		Rhizobiales	Brucellaceae	*Daeguia*
				
		Rhodobacterales	Rhodobacteraceae	*Gemmobacter*
	Deltaproteobacteria	Myxococcales		
				
Actinobacteria	Actinobacteria	Actinomycetales	Mycobacteriaceae	*Mycobacterium*
		Acidimicrobiales		
Chloroflexi	Caldilineae	Caldilineales	Caldineaceae	*Litorilinea*

## Supplementary Materials

The following are available online at https://www.mdpi.com/2076-2607/8/7/1040/s1, Figure S1: Differential abundance analysis of some phyla. Figure S2: Principal coordinate analysis (PCoA) plot of all samples between intestinal content and mucosa. Each dot represents one sample. Figure S3: Rarefaction curve, Table S1: Bacterial genus significantly different between lake Awassa and Chamo. Table S2: Bacterial genus significantly different between the gut regions. Table S3: Goods coverage.
