# Supplementary material for: Metabarcoding Analyses of Gut Microbiota of Nile Tilapia (Oreochromis niloticus) from Lake Awassa and Lake Chamo, Ethiopia
Source: Microorganisms. 2020 Jul 13;8(7):1040. doi: 10.3390/microorganisms8071040 (PMC7409238; doi:10.3390/microorganisms8071040)
Supplement: Supplementary file 1 [file microorganisms-08-01040-s001.pdf]

Supplementary materials

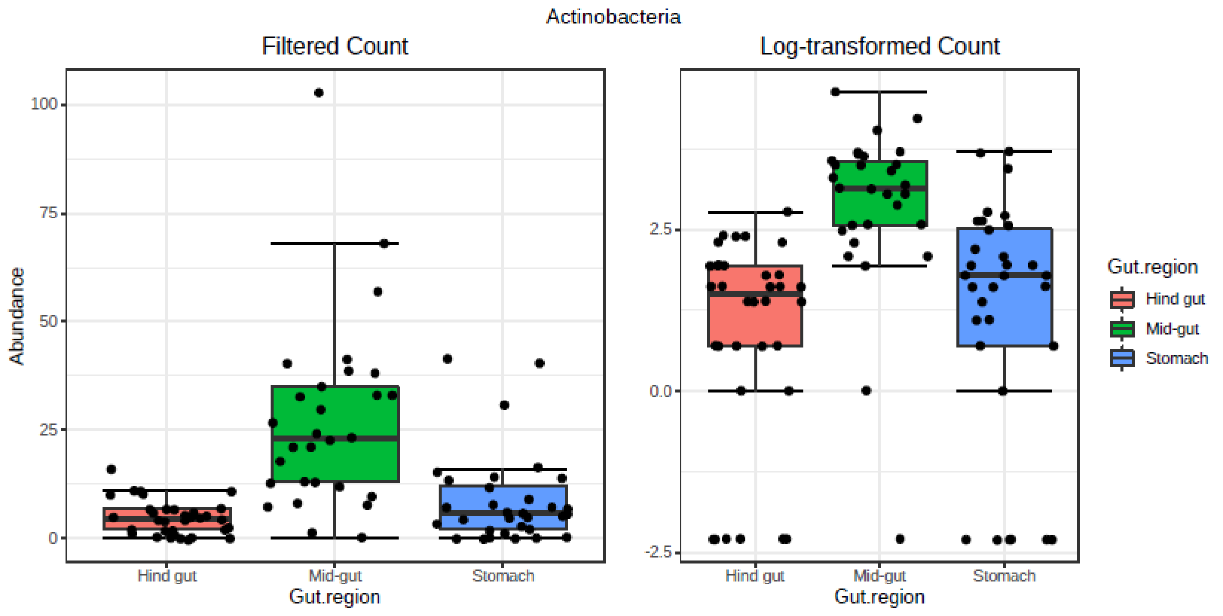

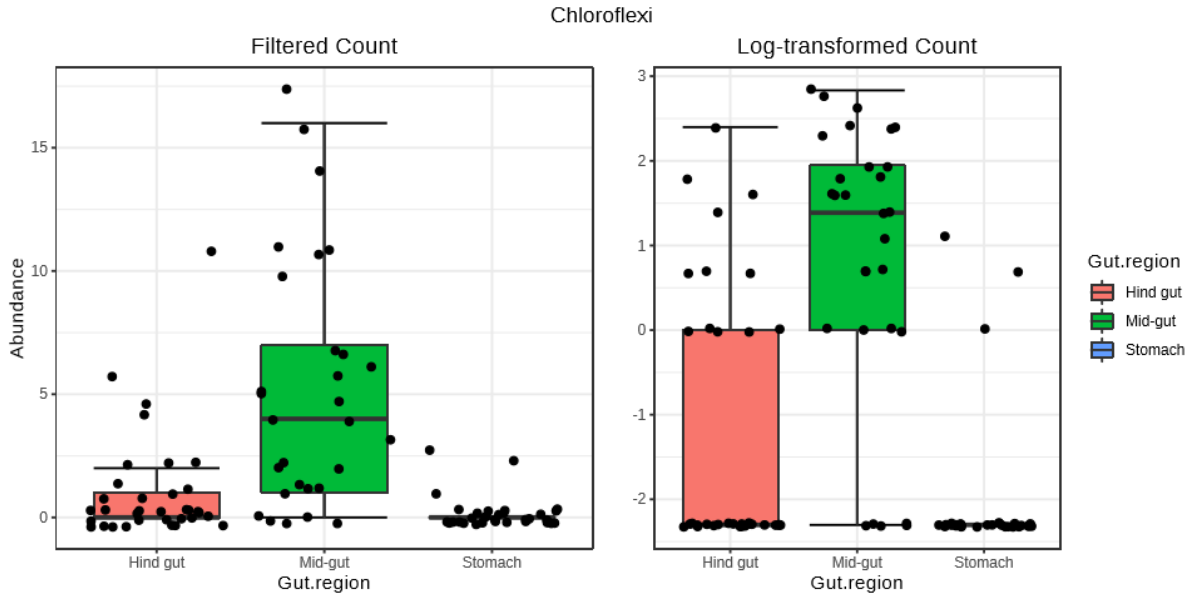

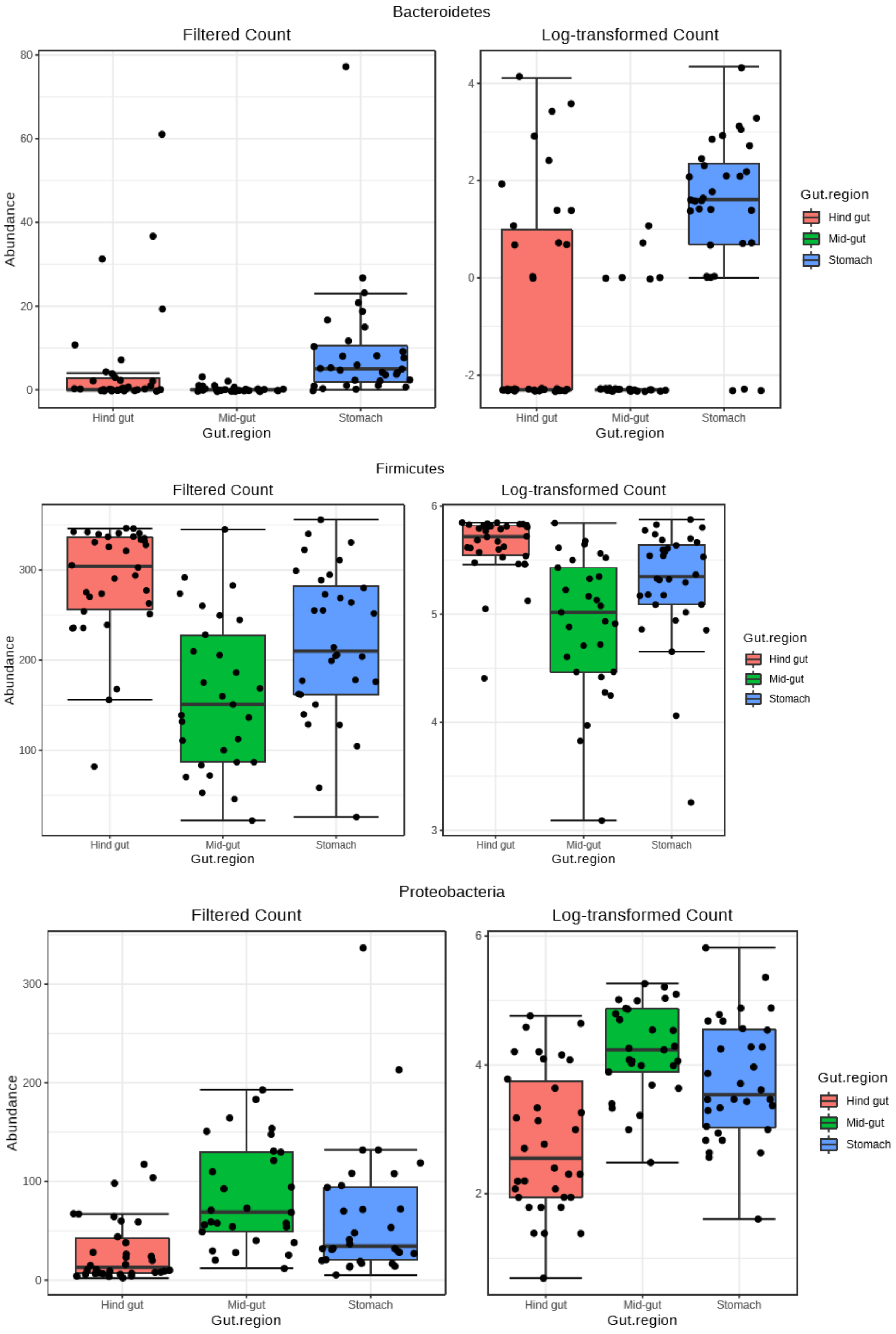
Bacteroidetes
Filtered Count
Log-transformed Count
Abundance
80
60
40
20
0
4
2
0
-2
Hind gut
Mid-gut
Stomach
Gut.region
Gut.region
Hind gut
Mid-gut
Stomach
Firmicutes
Filtered Count
Log-transformed Count
Abundance
300
200
100
6
5
4
3
Hind gut
Mid-gut
Stomach
Gut.region
Gut.region
Hind gut
Mid-gut
Stomach
Proteobacteria
Filtered Count
Log-transformed Count
Abundance
300
200
100
0
6
4
2
Hind gut
Mid-gut
Stomach
Gut.region
Gut.region
Hind gut
Mid-gut
Stomach

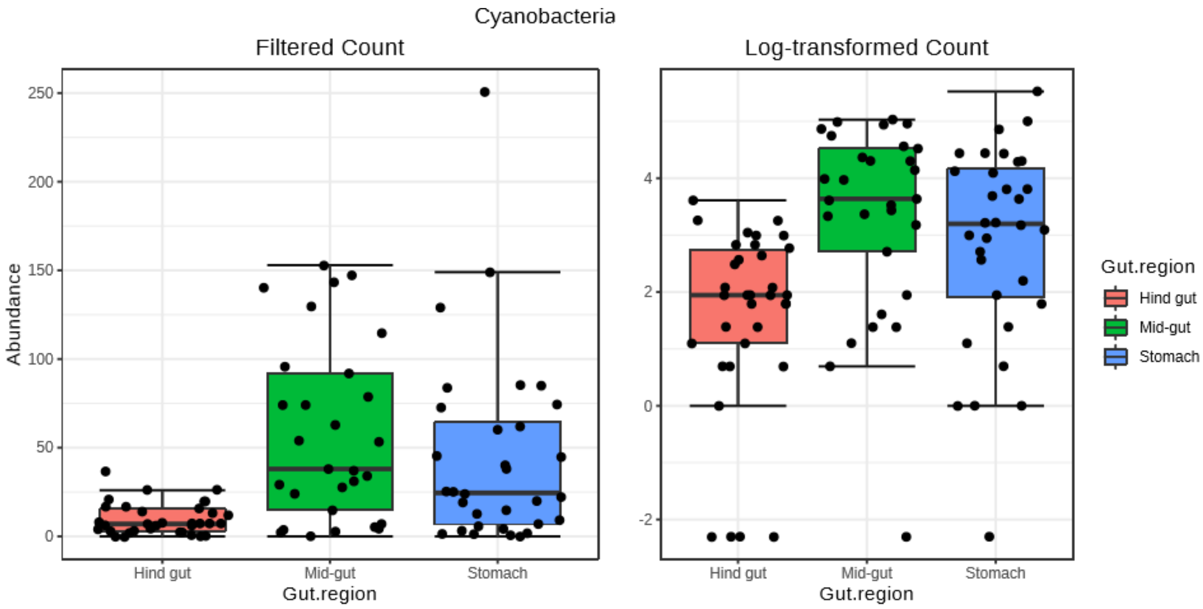

Supplementary figure1. Differential abundance analysis of some phyla.

Supplementary Table 1. Bacterial genus significantly different between lake Awassa and Chamo.

Genus	Pvalues	FDR	Statistics
Romboutsia	3.58E-13	1.50E-11	155.5
Orientia	1.40E-12	2.95E-11	1925
GpIIa	2.37E-12	3.32E-11	1957.5
Candidatus_Carsonella	4.28E-12	4.49E-11	1959.5
Aquihabitans	2.56E-10	2.15E-09	1800
Bacillus	1.78E-09	1.17E-08	326.5
Methyloparacoccus	1.94E-09	1.17E-08	348
Gemmobacter	2.99E-09	1.57E-08	461.5
Sporolactobacillaceae_incertae_sedis	5.38E-09	2.51E-08	495
Nocardioides	2.88E-08	1.21E-07	540
Cetobacterium	3.31E-08	1.26E-07	384
Lysinibacillus	3.79E-08	1.33E-07	530.5
Mycobacterium	1.43E-07	4.62E-07	1805.5
Oceanicola	2.89E-07	8.11E-07	607.5
Enhygromyxa	2.90E-07	8.11E-07	607.5
Micromonospora	8.20E-07	2.15E-06	599.5
Rhodobacter	9.42E-07	2.33E-06	622.5
Terrisporobacter	1.87E-06	4.36E-06	552
Rhodoplanes	2.57E-06	5.69E-06	675
Povalibacter	1.89E-05	3.96E-05	680
Bacillariophyta	3.07E-05	6.14E-05	1683
Daeguia	0.000147	0.000281	631.5
Clostridium_sensu_stricto	0.000318	0.000561	1608.5
Chlorophyta	0.00032	0.000561	1545
Aggregicoccus	0.000499	0.000839	1525.5
Olsenella	0.001355	0.002189	807
Ralstonia	0.001579	0.002456	1466.5

Flexithrix	0.003996	0.005994	1456.5
Cellulosilyticum	0.00662	0.009588	821.5
Turicibacter	0.01242	0.017388	1460.5
Paludibacter	0.013698	0.018559	865
GpXI	0.017986	0.023607	1439.5
Methylocystis	0.032206	0.04099	868.5
Rubrivivax	0.036066	0.044552	904

Supplementary table 2. Bacterial genus significantly different between the gut regions.

Genus	Pvalues	FDR	Statistics
Roseomonas	4.66E-12	1.96E-10	52.186
Aciditerrimonas	5.12E-11	1.08E-09	47.391
Clostridium_XI	6.32E-10	8.84E-09	42.365
Litorilinea	3.15E-09	3.31E-08	39.15
Clostridium_sensu_stricto	4.97E-08	4.17E-07	33.635
Daeguia	1.47E-07	1.02E-06	31.47
Flexithrix	1.71E-07	1.02E-06	31.166
Aggregicoccus	1.72E-06	9.04E-06	26.543
Bacillariophyta	2.56E-06	1.20E-05	25.749
Methylocystis	2.85E-06	1.20E-05	25.535
Mycobacterium	3.64E-06	1.39E-05	25.048
Terrisporobacter	8.61E-06	3.01E-05	23.325
Rubrivivax	1.49E-05	4.80E-05	22.235
Motilibacter	3.40E-05	0.000101	20.58
Thiolamprovum	3.59E-05	0.000101	20.469
Aquihabitans	0.000101	0.000264	18.409
Bacillus	0.000112	0.000276	18.196
Cellulosilyticum	0.000149	0.000344	17.619
Hyphomicrobium	0.000156	0.000344	17.536
GpXI	0.001019	0.00214	13.778
Enhygromyxa	0.001191	0.002382	13.466
Rhodoplanes	0.001359	0.002594	13.202
Povalibacter	0.002207	0.00403	12.233
Gemmobacter	0.00241	0.004217	12.056
Methyloparacoccus	0.004406	0.007402	10.85
Sporolactobacillaceae_incertae_sedis	0.006716	0.010849	10.007
Candidatus_Carsonella	0.00882	0.013719	9.4616
Paludibacter	0.010146	0.015219	9.1814
Turicibacter	0.01152	0.016684	8.9274
Olsenella	0.01353	0.018705	8.6056
Lysinibacillus	0.013806	0.018705	8.5653
Chlorophyta	0.03111	0.040831	6.9405
Romboutsia	0.033325	0.042413	6.8029
Ralstonia	0.037524	0.046353	6.5655
Orientia	0.039062	0.046522	6.4852
Oceanicola	0.039876	0.046522	6.444

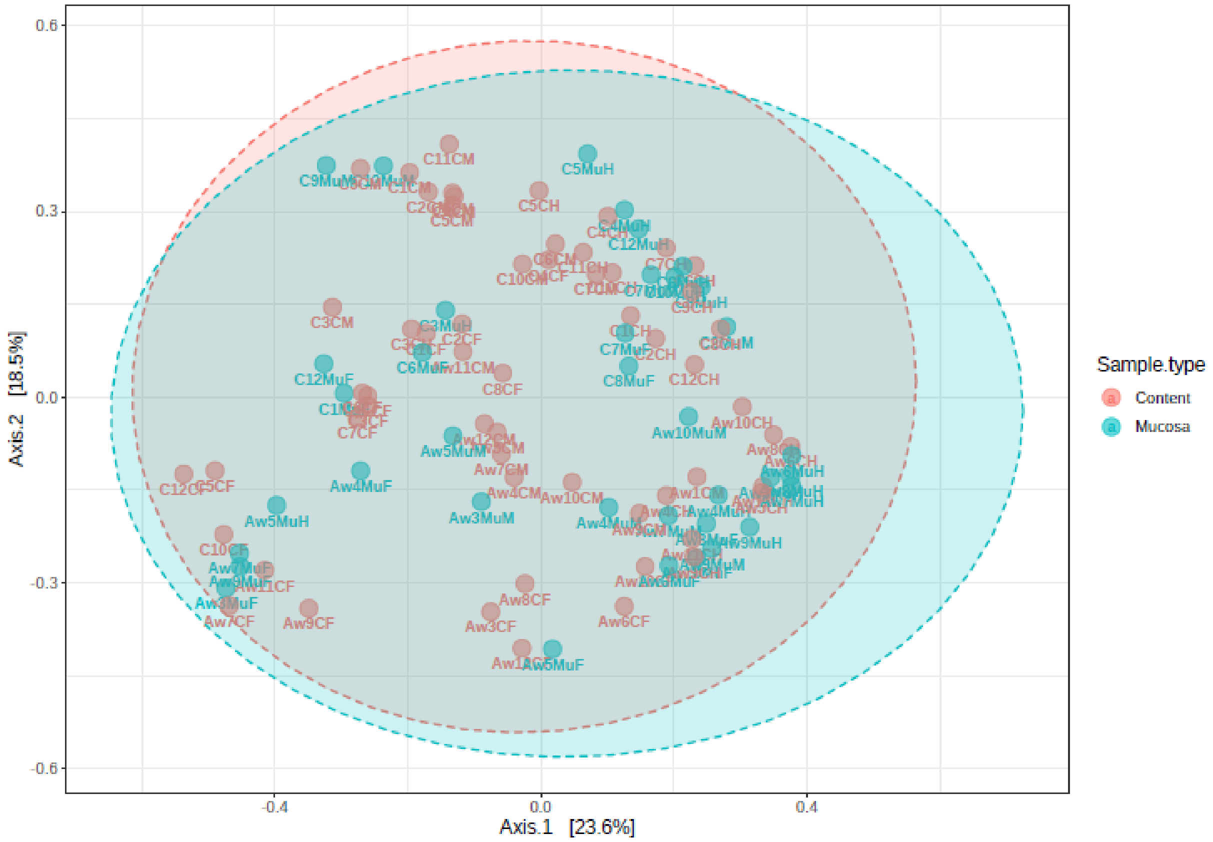

Suppementary figure 2: Principal coordinate analysis (PCoA) plot of all samples between intestinal content and mucosa. Each dot represents one sample.

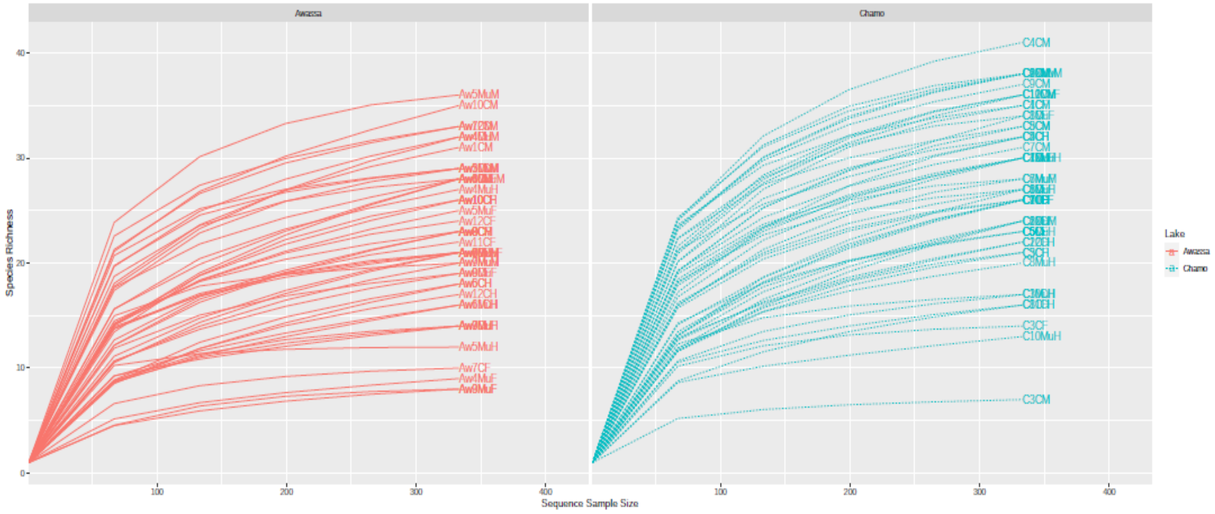

Supplementary figure 3: Rarefaction curve

Supplementary table 3: Goods coverage

Sample	Goods	Sample	Goods
Aw10CF	96.75676	C10MuH	99.18919
Aw10CH	98.64865	C11CF	97.2973
Aw10CM	97.56757	C11CH	99.18919
Aw10MuF	98.91892	C11CM	97.2973
Aw10MuM	99.18919	C12CF	98.10811
Aw11CF	98.37838	C12CH	99.18919
Aw11CH	98.37838	C12MuF	98.91892
Aw11CM	98.91892	C12MuH	98.10811
Aw12CF	98.64865	C12MuM	98.37838
Aw12CH	97.56757	C1CF	99.45946
Aw12CM	98.10811	C1CH	98.91892
Aw1CM	97.83784	C1CM	98.91892
Aw3CF	98.10811	C1MuF	97.83784
Aw3MuF	99.45946	C2CF	98.64865
Aw3MuH	98.37838	C2CH	98.64865
Aw3MuM	99.18919	C2CM	98.37838
Aw4CH	97.56757	C2MuM	97.2973
Aw4CM	98.37838	C3CF	98.64865
Aw4MuF	98.91892	C3CH	98.64865
Aw4MuH	98.37838	C3CM	99.45946
Aw4MuM	98.10811	C3MuH	99.45946
Aw5CH	98.10811	C4CF	98.37838
Aw5CM	99.18919	C4CH	97.02703
Aw5MuF	98.10811	C4CM	98.91892
Aw5MuH	100	C4MuH	97.56757
Aw5MuM	98.37838	C5CF	98.64865
Aw6CF	98.91892	C5CH	97.83784
Aw6CH	98.64865	C5CM	99.18919

Aw6MuF	98.10811	C5MuH	98.10811
Aw6MuH	99.45946	C6CF	98.10811
Aw7CF	99.18919	C6CH	97.2973
Aw7CM	98.37838	C6CM	98.91892
Aw7MuF	98.37838	C6MuF	97.56757
Aw7MuH	98.10811	C6MuH	98.37838
Aw7MuM	99.72973	C7CF	99.45946
Aw8CF	97.83784	C7CH	98.64865
Aw8CH	97.83784	C7CM	98.91892
Aw8MuF	98.64865	C7MuF	97.83784
Aw8MuH	97.83784	C7MuM	98.64865
Aw9CF	99.18919	C8CF	98.91892
Aw9CH	98.91892	C8CH	97.83784
Aw9CM	97.83784	C8CM	99.72973
Aw9MuF	99.72973	C8MuF	97.02703
Aw9MuH	97.56757	C8MuH	98.91892
Aw9MuM	98.91892	C9CH	97.83784
C10CF	98.37838	C9CM	98.37838
C10CH	99.45946	C9MuM	98.10811
C10CM	97.56757		
